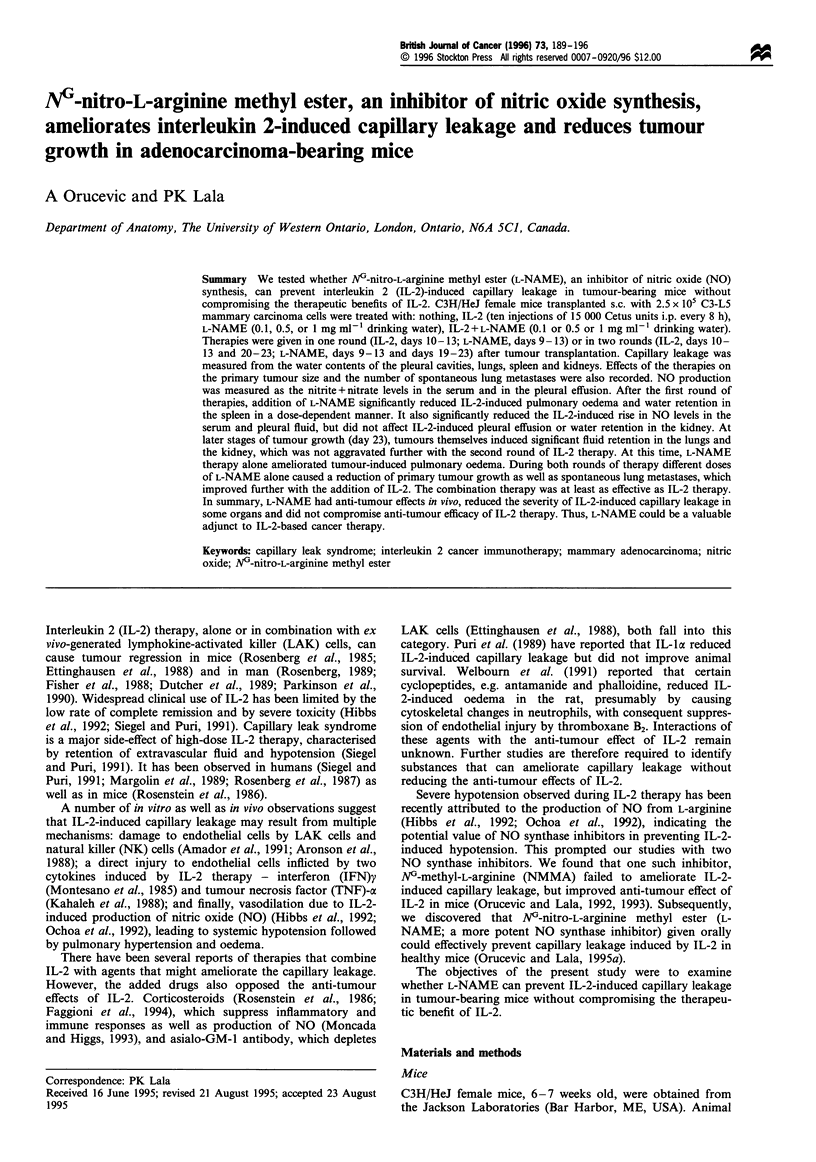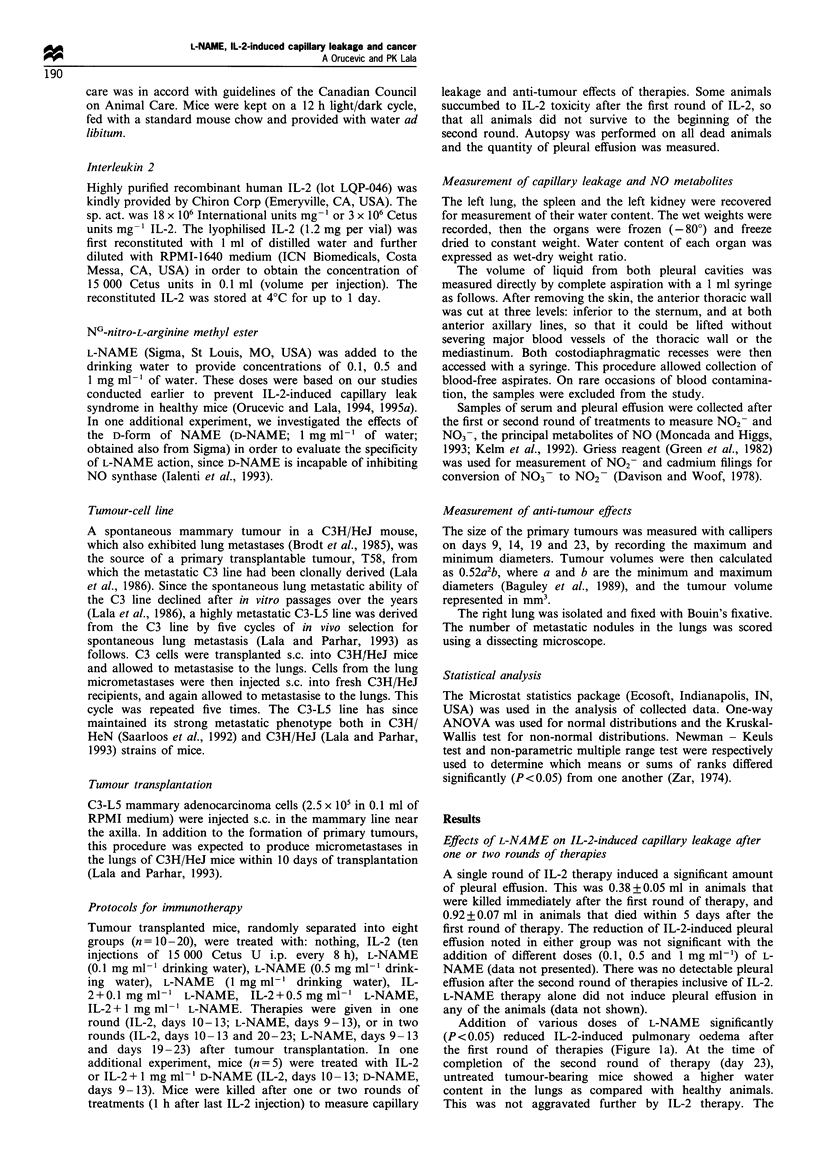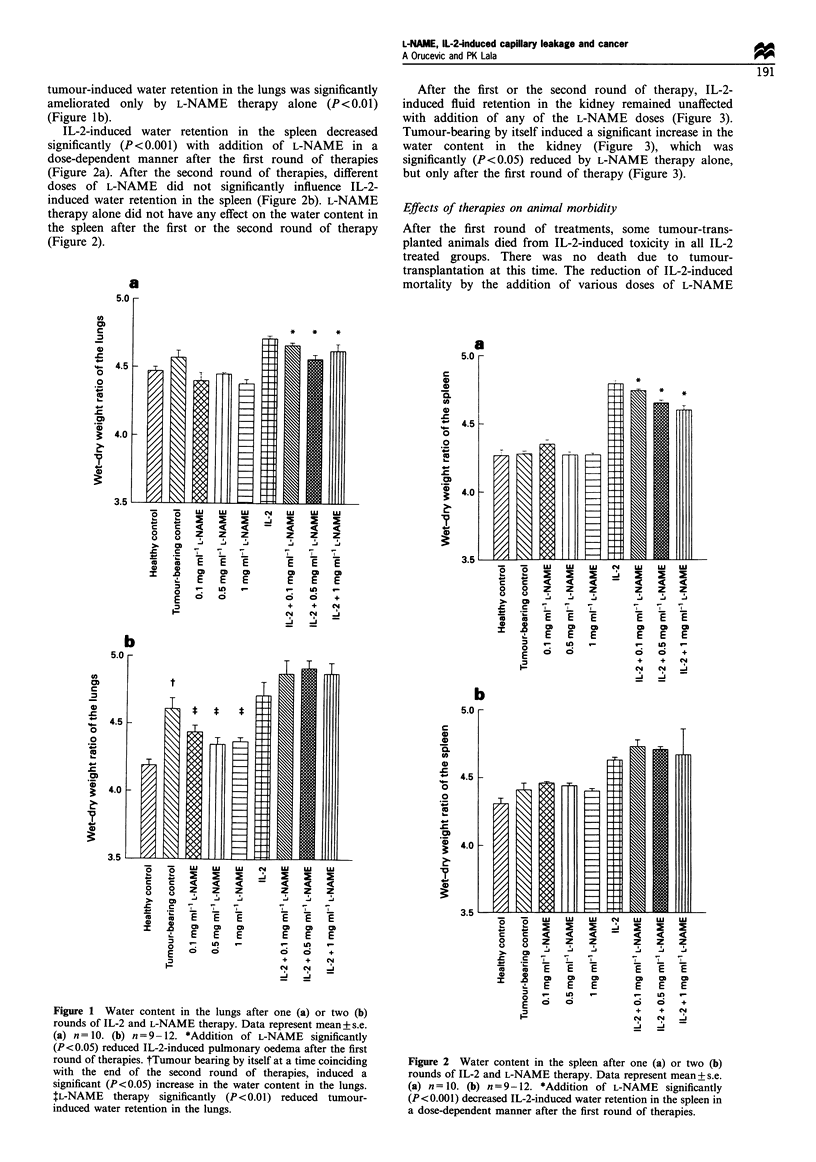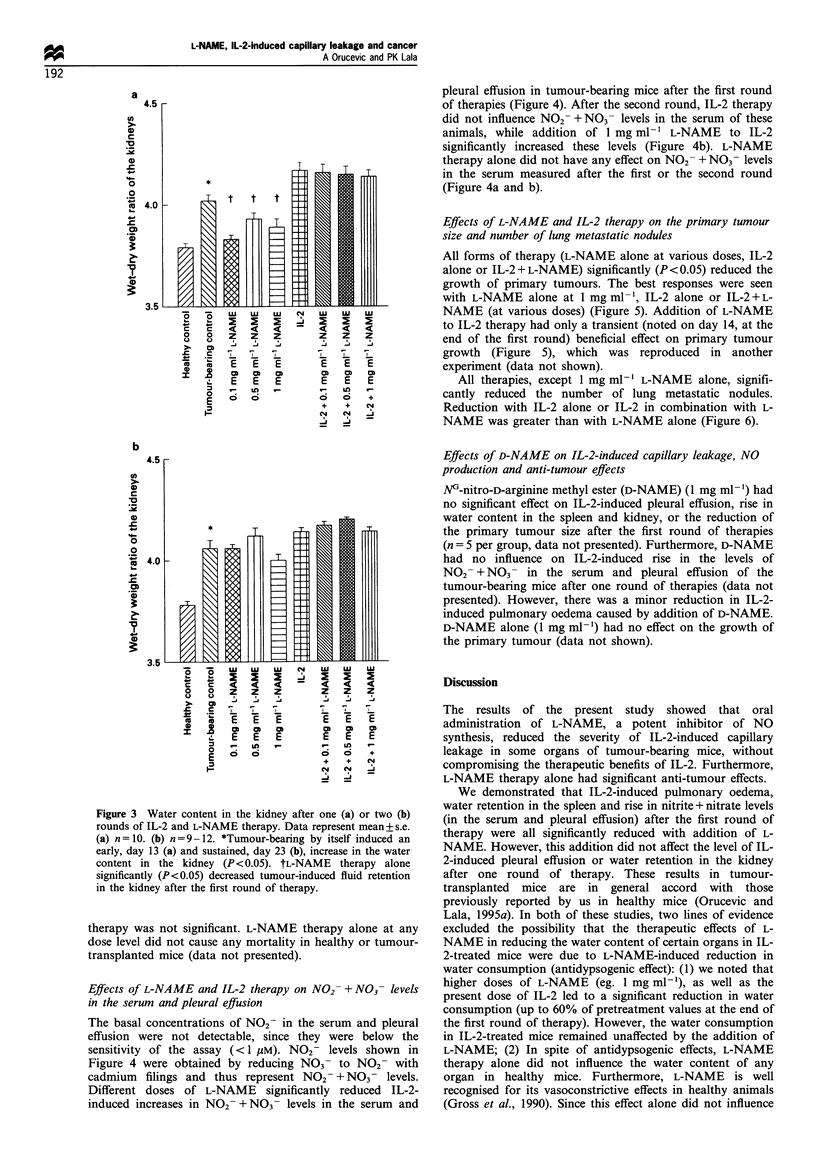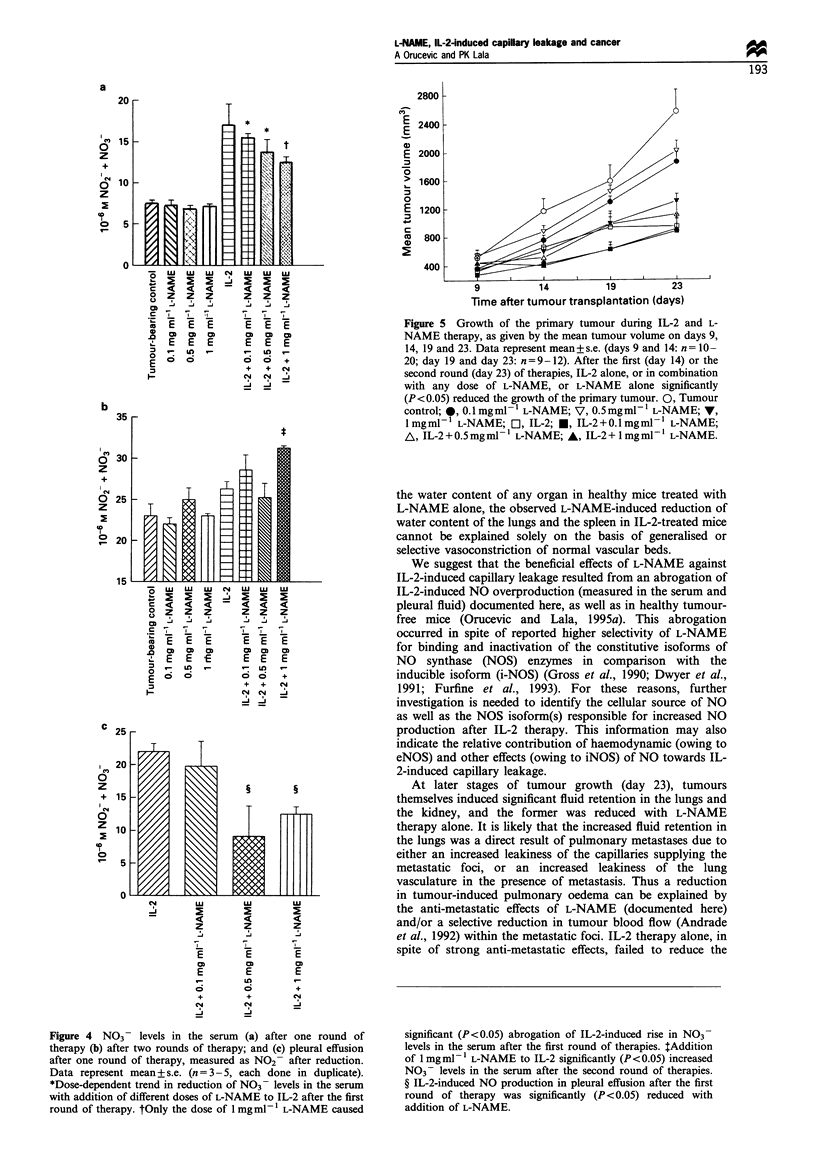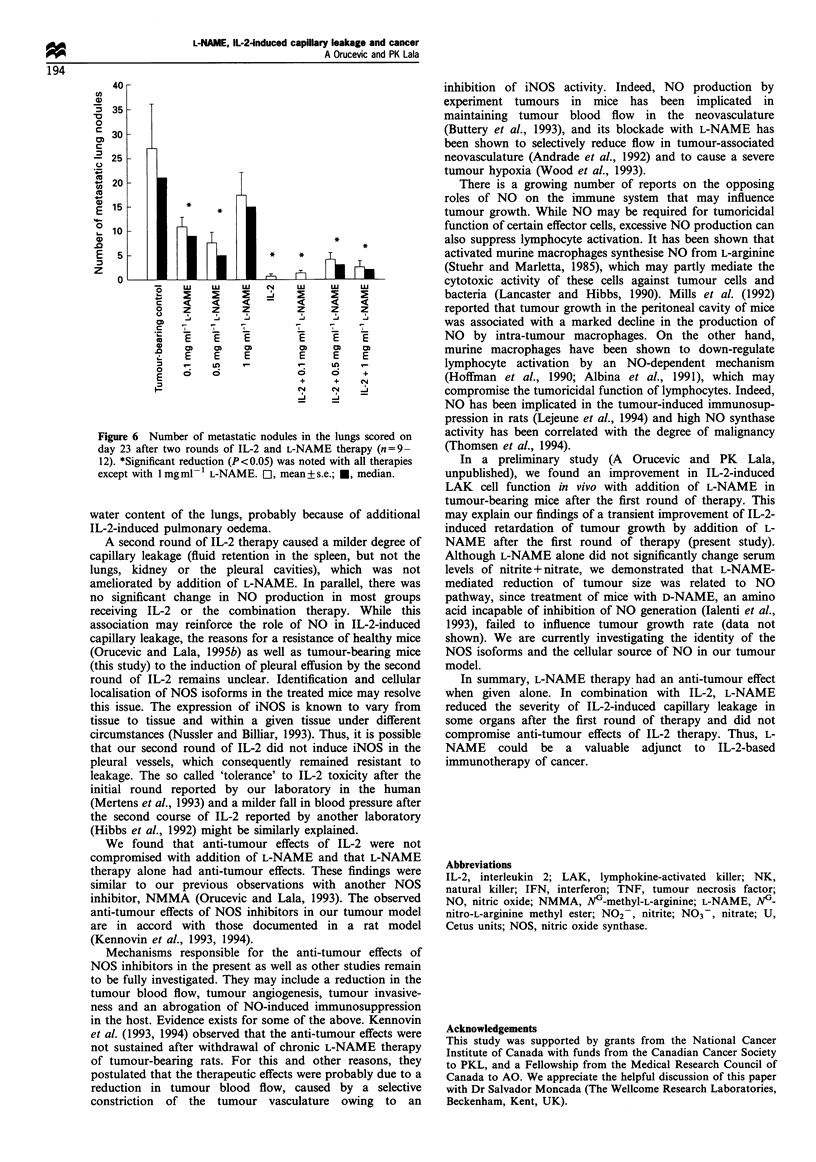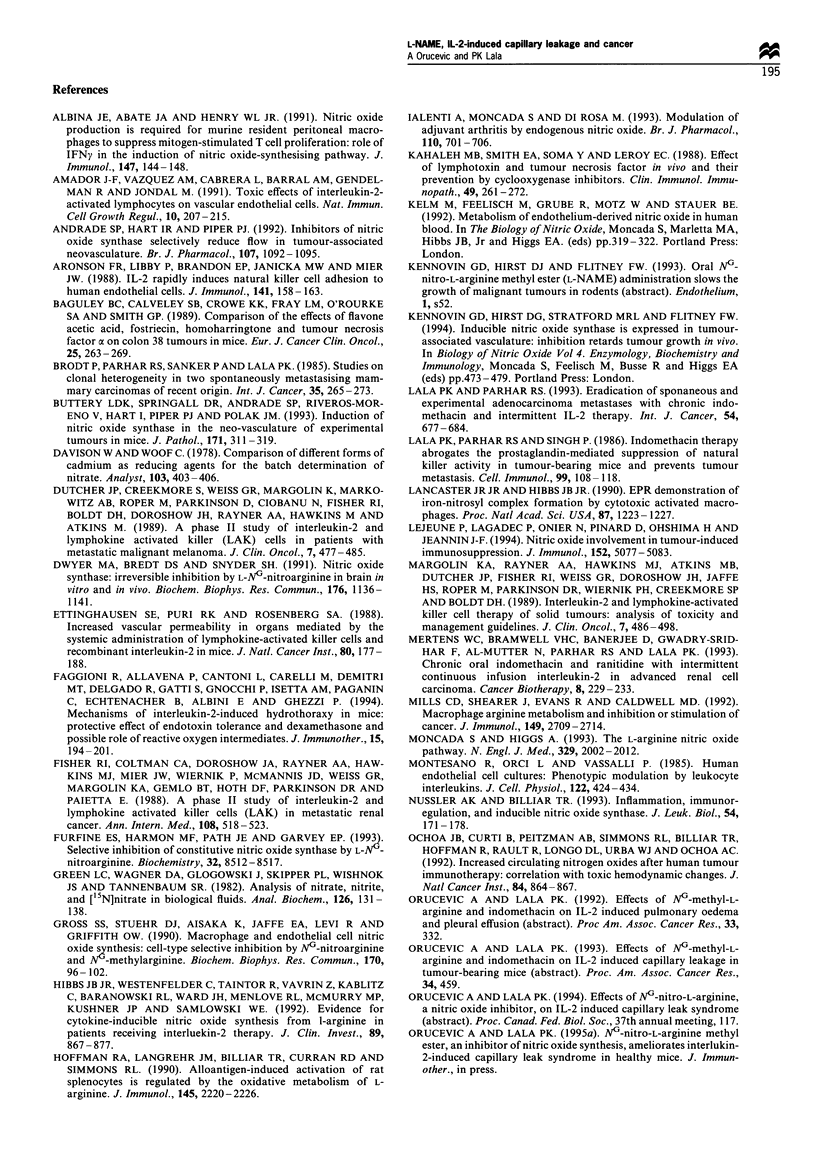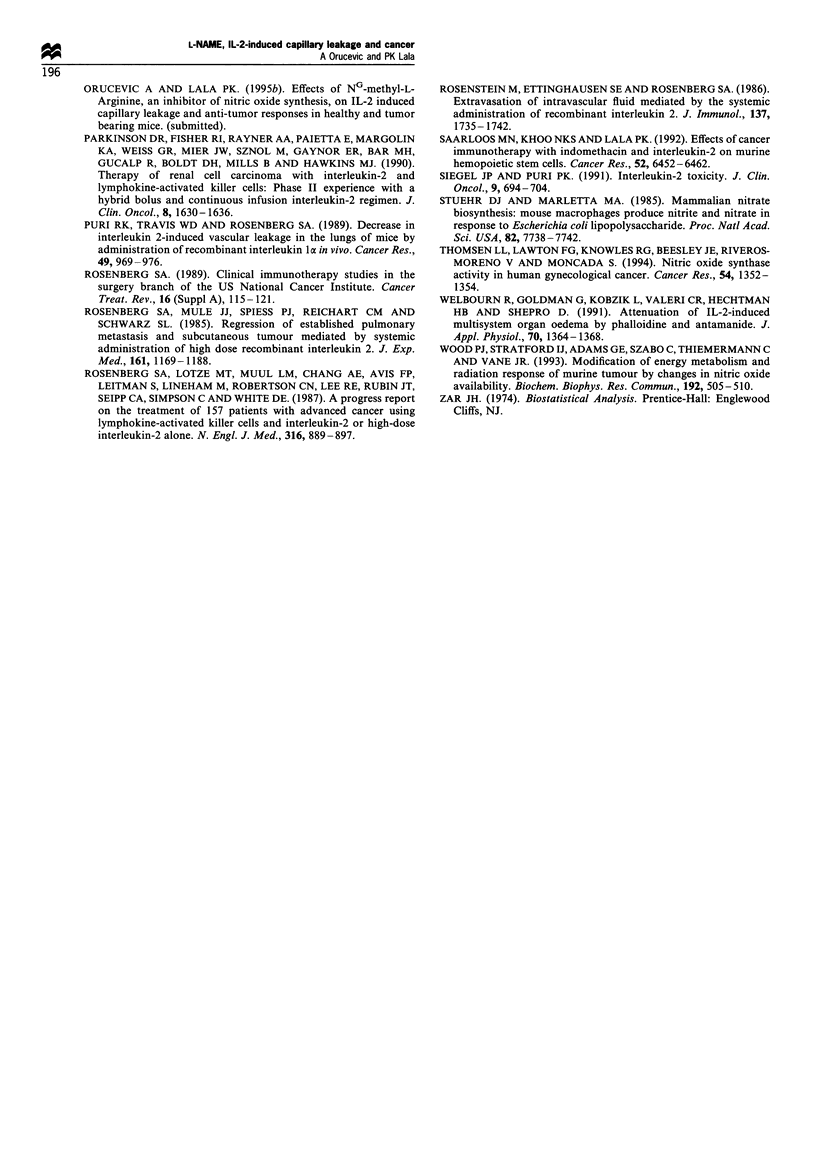# NG-nitro-L-arginine methyl ester, an inhibitor of nitric oxide synthesis, ameliorates interleukin 2-induced capillary leakage and reduces tumour growth in adenocarcinoma-bearing mice.

**DOI:** 10.1038/bjc.1996.34

**Published:** 1996-01

**Authors:** A. Orucevic, P. K. Lala

**Affiliations:** Department of Anatomy, University of Western Ontario, London, Canada.

## Abstract

We tested whether NG-nitro-L-arginine methyl ester (L-NAME), an inhibitor of nitric oxide (NO) synthesis, can prevent interleukin 2 (IL-2)-induced capillary leakage in tumour-bearing mice without compromising the therapeutic benefits of IL-2. C3H/HeJ female mice transplanted s.c. with 2.5 x 10(5) C3-L5 mammary carcinoma cells were treated with: nothing, IL-2 (ten injections of 15,000 Cetus units i.p. every 8 h), L-NAME (0.1, 0.5, or 1 mg ml-1 drinking water), IL-2 + L-NAME (0.1 or 0.5 or 1 mg ml-1 drinking water). Therapies were given in one round (IL-2, days 10-13; L-NAME, days 9-13) or in two rounds (IL-2, days 10-13 and 20-23; L-NAME, days 9-13 and days 19-23) after tumour transplantation. Capillary leakage was measured from the water contents of the pleural cavities, lungs, spleen and kidneys. Effects of the therapies on the primary tumour size and the number of spontaneous lung metastases were also recorded. NO production was measured as the nitrite + nitrate levels in the serum and in the pleural effusion. After the first round of therapies, addition of L-NAME significantly reduced IL-2-induced pulmonary oedema and water retention in the spleen in a dose-dependent manner. It also significantly reduced the IL-2-induced rise in NO levels in the serum and pleural fluid, but did not affect IL-2-induced pleural effusion or water retention in the kidney. At later stages of tumour growth (day 23), tumours themselves induced significant fluid retention in the lungs and the kidney, which was not aggravated further with the second round of IL-2 therapy. At this time, L-NAME therapy alone ameliorated tumour-induced pulmonary oedema. During both rounds of therapy different doses of L-NAME alone caused a reduction of primary tumour growth as well as spontaneous lung metastases, which improved further with the addition of IL-2. The combination therapy was at least as effective as IL-2 therapy. In summary, L-NAME had anti-tumour effects in vivo, reduced the severity of IL-2-induced capillary leakage in some organs and did not compromise anti-tumour efficacy of IL-2 therapy. Thus, L-NAME could be a valuable adjunct to IL-2-based cancer therapy.


British Journal of Cancer (1996) 73, 189-196
© 1996 Stockton Press All rights reserved 0007-0920/96 $12.00

### $N^G$-nitro-L-arginine methyl ester, an inhibitor of nitric oxide synthesis, ameliorates interleukin 2-induced capillary leakage and reduces tumour growth in adenocarcinoma-bearing mice

A Orucevic and PK Lala

*Department of Anatomy, The University of Western Ontario, London, Ontario, N6A 5C1, Canada.*

**Summary** We tested whether $N^G$-nitro-L-arginine methyl ester (L-NAME), an inhibitor of nitric oxide (NO) synthesis, can prevent interleukin 2 (IL-2)-induced capillary leakage in tumour-bearing mice without compromising the therapeutic benefits of IL-2. C3H/HeJ female mice transplanted s.c. with $2.5 \times 10^5$ C3-L5 mammary carcinoma cells were treated with: nothing, IL-2 (ten injections of 15 000 Cetus units i.p. every 8 h), L-NAME (0.1, 0.5, or 1 mg $ml^{-1}$ drinking water), IL-2 + L-NAME (0.1 or 0.5 or 1 mg $ml^{-1}$ drinking water). Therapies were given in one round (IL-2, days 10–13; L-NAME, days 9–13) or in two rounds (IL-2, days 10–13 and 20–23; L-NAME, days 9–13 and days 19–23) after tumour transplantation. Capillary leakage was measured from the water contents of the pleural cavities, lungs, spleen and kidneys. Effects of the therapies on the primary tumour size and the number of spontaneous lung metastases were also recorded. NO production was measured as the nitrite + nitrate levels in the serum and in the pleural effusion. After the first round of therapies, addition of L-NAME significantly reduced IL-2-induced pulmonary oedema and water retention in the spleen in a dose-dependent manner. It also significantly reduced the IL-2-induced rise in NO levels in the serum and pleural fluid, but did not affect IL-2-induced pleural effusion or water retention in the kidney. At later stages of tumour growth (day 23), tumours themselves induced significant fluid retention in the lungs and the kidney, which was not aggravated further with the second round of IL-2 therapy. At this time, L-NAME therapy alone ameliorated tumour-induced pulmonary oedema. During both rounds of therapy different doses of L-NAME alone caused a reduction of primary tumour growth as well as spontaneous lung metastases, which improved further with the addition of IL-2. The combination therapy was at least as effective as IL-2 therapy. In summary, L-NAME had anti-tumour effects *in vivo*, reduced the severity of IL-2-induced capillary leakage in some organs and did not compromise anti-tumour efficacy of IL-2 therapy. Thus, L-NAME could be a valuable adjunct to IL-2-based cancer therapy.

**Keywords:** capillary leak syndrome; interleukin 2 cancer immunotherapy; mammary adenocarcinoma; nitric oxide; $N^G$-nitro-L-arginine methyl ester

Interleukin 2 (IL-2) therapy, alone or in combination with *ex vivo*-generated lymphokine-activated killer (LAK) cells, can cause tumour regression in mice (Rosenberg *et al.*, 1985; Ettinghausen *et al.*, 1988) and in man (Rosenberg, 1989; Fisher *et al.*, 1988; Dutcher *et al.*, 1989; Parkinson *et al.*, 1990). Widespread clinical use of IL-2 has been limited by the low rate of complete remission and by severe toxicity (Hibbs *et al.*, 1992; Siegel and Puri, 1991). Capillary leak syndrome is a major side-effect of high-dose IL-2 therapy, characterised by retention of extravascular fluid and hypotension (Siegel and Puri, 1991). It has been observed in humans (Siegel and Puri, 1991; Margolin *et al.*, 1989; Rosenberg *et al.*, 1987) as well as in mice (Rosenstein *et al.*, 1986).

A number of *in vitro* as well as *in vivo* observations suggest that IL-2-induced capillary leakage may result from multiple mechanisms: damage to endothelial cells by LAK cells and natural killer (NK) cells (Amador *et al.*, 1991; Aronson *et al.*, 1988); a direct injury to endothelial cells inflicted by two cytokines induced by IL-2 therapy – interferon (IFN)$\gamma$ (Montesano *et al.*, 1985) and tumour necrosis factor (TNF)-$\alpha$ (Kahaleh *et al.*, 1988); and finally, vasodilation due to IL-2-induced production of nitric oxide (NO) (Hibbs *et al.*, 1992; Ochoa *et al.*, 1992), leading to systemic hypotension followed by pulmonary hypertension and oedema.

There have been several reports of therapies that combine IL-2 with agents that might ameliorate the capillary leakage. However, the added drugs also opposed the anti-tumour effects of IL-2. Corticosteroids (Rosenstein *et al.*, 1986; Faggioni *et al.*, 1994), which suppress inflammatory and immune responses as well as production of NO (Moncada and Higgs, 1993), and asialo-GM-1 antibody, which depletes LAK cells (Ettinghausen *et al.*, 1988), both fall into this category. Puri *et al.* (1989) have reported that IL-1$\alpha$ reduced IL-2-induced capillary leakage but did not improve animal survival. Welbourn *et al.* (1991) reported that certain cyclopeptides, e.g. antamanide and phalloidine, reduced IL-2-induced oedema in the rat, presumably by causing cytoskeletal changes in neutrophils, with consequent suppression of endothelial injury by thromboxane $B_2$. Interactions of these agents with the anti-tumour effect of IL-2 remain unknown. Further studies are therefore required to identify substances that can ameliorate capillary leakage without reducing the anti-tumour effects of IL-2.

Severe hypotension observed during IL-2 therapy has been recently attributed to the production of NO from L-arginine (Hibbs *et al.*, 1992; Ochoa *et al.*, 1992), indicating the potential value of NO synthase inhibitors in preventing IL-2-induced hypotension. This prompted our studies with two NO synthase inhibitors. We found that one such inhibitor, $N^G$-methyl-L-arginine (NMMA) failed to ameliorate IL-2-induced capillary leakage, but improved anti-tumour effect of IL-2 in mice (Orucevic and Lala, 1992, 1993). Subsequently, we discovered that $N^G$-nitro-L-arginine methyl ester (L-NAME; a more potent NO synthase inhibitor) given orally could effectively prevent capillary leakage induced by IL-2 in healthy mice (Orucevic and Lala, 1995*a*).

The objectives of the present study were to examine whether L-NAME can prevent IL-2-induced capillary leakage in tumour-bearing mice without compromising the therapeutic benefit of IL-2.

### Materials and methods

### *Mice*

C3H/HeJ female mice, 6–7 weeks old, were obtained from the Jackson Laboratories (Bar Harbor, ME, USA). Animal

Correspondence: PK Lala
Received 16 June 1995; revised 21 August 1995; accepted 23 August 1995

care was in accord with guidelines of the Canadian Council on Animal Care. Mice were kept on a 12 h light/dark cycle, fed with a standard mouse chow and provided with water *ad libitum*.

*Interleukin 2*

Highly purified recombinant human IL-2 (lot LQP-046) was kindly provided by Chiron Corp (Emeryville, CA, USA). The sp. act. was $18 \times 10^6$ International units $mg^{-1}$ or $3 \times 10^6$ Cetus units $mg^{-1}$ IL-2. The lyophilised IL-2 (1.2 mg per vial) was first reconstituted with 1 ml of distilled water and further diluted with RPMI-1640 medium (ICN Biomedicals, Costa Messa, CA, USA) in order to obtain the concentration of 15 000 Cetus units in 0.1 ml (volume per injection). The reconstituted IL-2 was stored at 4°C for up to 1 day.

*$N^G$-nitro-L-arginine methyl ester*

L-NAME (Sigma, St Louis, MO, USA) was added to the drinking water to provide concentrations of 0.1, 0.5 and 1 mg $ml^{-1}$ of water. These doses were based on our studies conducted earlier to prevent IL-2-induced capillary leak syndrome in healthy mice (Orucevic and Lala, 1994, 1995*a*). In one additional experiment, we investigated the effects of the D-form of NAME (D-NAME; 1 mg $ml^{-1}$ of water; obtained also from Sigma) in order to evaluate the specificity of L-NAME action, since D-NAME is incapable of inhibiting NO synthase (Ialenti *et al.*, 1993).

*Tumour-cell line*

A spontaneous mammary tumour in a C3H/HeJ mouse, which also exhibited lung metastases (Brodt *et al.*, 1985), was the source of a primary transplantable tumour, T58, from which the metastatic C3 line had been clonally derived (Lala *et al.*, 1986). Since the spontaneous lung metastatic ability of the C3 line declined after *in vitro* passages over the years (Lala *et al.*, 1986), a highly metastatic C3-L5 line was derived from the C3 line by five cycles of *in vivo* selection for spontaneous lung metastasis (Lala and Parhar, 1993) as follows. C3 cells were transplanted s.c. into C3H/HeJ mice and allowed to metastasise to the lungs. Cells from the lung micrometastases were then injected s.c. into fresh C3H/HeJ recipients, and again allowed to metastasise to the lungs. This cycle was repeated five times. The C3-L5 line has since maintained its strong metastatic phenotype both in C3H/HeN (Saarloos *et al.*, 1992) and C3H/HeJ (Lala and Parhar, 1993) strains of mice.

*Tumour transplantation*

C3-L5 mammary adenocarcinoma cells ($2.5 \times 10^5$ in 0.1 ml of RPMI medium) were injected s.c. in the mammary line near the axilla. In addition to the formation of primary tumours, this procedure was expected to produce micrometastases in the lungs of C3H/HeJ mice within 10 days of transplantation (Lala and Parhar, 1993).

*Protocols for immunotherapy*

Tumour transplanted mice, randomly separated into eight groups ($n = 10-20$), were treated with: nothing, IL-2 (ten injections of 15 000 Cetus U i.p. every 8 h), L-NAME (0.1 mg $ml^{-1}$ drinking water), L-NAME (0.5 mg $ml^{-1}$ drinking water), L-NAME (1 mg $ml^{-1}$ drinking water), IL-2 + 0.1 mg $ml^{-1}$ L-NAME, IL-2 + 0.5 mg $ml^{-1}$ L-NAME, IL-2 + 1 mg $ml^{-1}$ L-NAME. Therapies were given in one round (IL-2, days 10–13; L-NAME, days 9–13), or in two rounds (IL-2, days 10–13 and 20–23; L-NAME, days 9–13 and days 19–23) after tumour transplantation. In one additional experiment, mice ($n = 5$) were treated with IL-2 or IL-2 + 1 mg $ml^{-1}$ D-NAME (IL-2, days 10–13; D-NAME, days 9–13). Mice were killed after one or two rounds of treatments (1 h after last IL-2 injection) to measure capillary leakage and anti-tumour effects of therapies. Some animals succumbed to IL-2 toxicity after the first round of IL-2, so that all animals did not survive to the beginning of the second round. Autopsy was performed on all dead animals and the quantity of pleural effusion was measured.

*Measurement of capillary leakage and NO metabolites*

The left lung, the spleen and the left kidney were recovered for measurement of their water content. The wet weights were recorded, then the organs were frozen (−80°) and freeze dried to constant weight. Water content of each organ was expressed as wet-dry weight ratio.

The volume of liquid from both pleural cavities was measured directly by complete aspiration with a 1 ml syringe as follows. After removing the skin, the anterior thoracic wall was cut at three levels: inferior to the sternum, and at both anterior axillary lines, so that it could be lifted without severing major blood vessels of the thoracic wall or the mediastinum. Both costodiaphragmatic recesses were then accessed with a syringe. This procedure allowed collection of blood-free aspirates. On rare occasions of blood contamination, the samples were excluded from the study.

Samples of serum and pleural effusion were collected after the first or second round of treatments to measure $NO_2^-$ and $NO_3^-$, the principal metabolites of NO (Moncada and Higgs, 1993; Kelm *et al.*, 1992). Griess reagent (Green *et al.*, 1982) was used for measurement of $NO_2^-$ and cadmium filings for conversion of $NO_3^-$ to $NO_2^-$ (Davison and Woof, 1978).

*Measurement of anti-tumour effects*

The size of the primary tumours was measured with callipers on days 9, 14, 19 and 23, by recording the maximum and minimum diameters. Tumour volumes were then calculated as $0.52a^2b$, where $a$ and $b$ are the minimum and maximum diameters (Baguley *et al.*, 1989), and the tumour volume represented in $mm^3$.

The right lung was isolated and fixed with Bouin's fixative. The number of metastatic nodules in the lungs was scored using a dissecting microscope.

*Statistical analysis*

The Microstat statistics package (Ecosoft, Indianapolis, IN, USA) was used in the analysis of collected data. One-way ANOVA was used for normal distributions and the Kruskal-Wallis test for non-normal distributions. Newman – Keuls test and non-parametric multiple range test were respectively used to determine which means or sums of ranks differed significantly ($P < 0.05$) from one another (Zar, 1974).

### Results

*Effects of L-NAME on IL-2-induced capillary leakage after one or two rounds of therapies*

A single round of IL-2 therapy induced a significant amount of pleural effusion. This was $0.38 \pm 0.05$ ml in animals that were killed immediately after the first round of therapy, and $0.92 \pm 0.07$ ml in animals that died within 5 days after the first round of therapy. The reduction of IL-2-induced pleural effusion noted in either group was not significant with the addition of different doses (0.1, 0.5 and 1 mg $ml^{-1}$) of L-NAME (data not presented). There was no detectable pleural effusion after the second round of therapies inclusive of IL-2. L-NAME therapy alone did not induce pleural effusion in any of the animals (data not shown).

Addition of various doses of L-NAME significantly ($P < 0.05$) reduced IL-2-induced pulmonary oedema after the first round of therapies (Figure 1a). At the time of completion of the second round of therapy (day 23), untreated tumour-bearing mice showed a higher water content in the lungs as compared with healthy animals. This was not aggravated further by IL-2 therapy. The tumour-induced water retention in the lungs was significantly ameliorated only by L-NAME therapy alone ($P<0.01$) (Figure 1b).

IL-2-induced water retention in the spleen decreased significantly ($P<0.001$) with addition of L-NAME in a dose-dependent manner after the first round of therapies (Figure 2a). After the second round of therapies, different doses of L-NAME did not significantly influence IL-2-induced water retention in the spleen (Figure 2b). L-NAME therapy alone did not have any effect on the water content in the spleen after the first or the second round of therapy (Figure 2).

**Figure 1** Water content in the lungs after one (**a**) or two (**b**) rounds of IL-2 and L-NAME therapy. Data represent mean ± s.e. (**a**) $n=10$. (**b**) $n=9-12$. *Addition of L-NAME significantly ($P<0.05$) reduced IL-2-induced pulmonary oedema after the first round of therapies. †Tumour bearing by itself at a time coinciding with the end of the second round of therapies, induced a significant ($P<0.05$) increase in the water content in the lungs. ‡L-NAME therapy significantly ($P<0.01$) reduced tumour-induced water retention in the lungs.

After the first or the second round of therapy, IL-2-induced fluid retention in the kidney remained unaffected with addition of any of the L-NAME doses (Figure 3). Tumour-bearing by itself induced a significant increase in the water content in the kidney (Figure 3), which was significantly ($P<0.05$) reduced by L-NAME therapy alone, but only after the first round of therapy (Figure 3).

*Effects of therapies on animal morbidity*

After the first round of treatments, some tumour-transplanted animals died from IL-2-induced toxicity in all IL-2 treated groups. There was no death due to tumour-transplantation at this time. The reduction of IL-2-induced mortality by the addition of various doses of L-NAME

**Figure 2** Water content in the spleen after one (**a**) or two (**b**) rounds of IL-2 and L-NAME therapy. Data represent mean ± s.e. (**a**) $n=10$. (**b**) $n=9-12$. *Addition of L-NAME significantly ($P<0.001$) decreased IL-2-induced water retention in the spleen in a dose-dependent manner after the first round of therapies.

**Figure 3** Water content in the kidney after one (**a**) or two (**b**) rounds of IL-2 and L-NAME therapy. Data represent mean ± s.e. (**a**) $n=10$. (**b**) $n=9-12$. *Tumour-bearing by itself induced an early, day 13 (**a**) and sustained, day 23 (**b**), increase in the water content in the kidney ($P<0.05$). †L-NAME therapy alone significantly ($P<0.05$) decreased tumour-induced fluid retention in the kidney after the first round of therapy.

therapy was not significant. L-NAME therapy alone at any dose level did not cause any mortality in healthy or tumour-transplanted mice (data not presented).

### *Effects of L-NAME and IL-2 therapy on $NO_2^-+NO_3^-$ levels in the serum and pleural effusion*

The basal concentrations of $NO_2^-$ in the serum and pleural effusion were not detectable, since they were below the sensitivity of the assay (<1 $\mu$M). $NO_2^-$ levels shown in Figure 4 were obtained by reducing $NO_3^-$ to $NO_2^-$ with cadmium filings and thus represent $NO_2^-+NO_3^-$ levels. Different doses of L-NAME significantly reduced IL-2-induced increases in $NO_2^-+NO_3^-$ levels in the serum and pleural effusion in tumour-bearing mice after the first round of therapies (Figure 4). After the second round, IL-2 therapy did not influence $NO_2^-+NO_3^-$ levels in the serum of these animals, while addition of 1 mg ml$^{-1}$ L-NAME to IL-2 significantly increased these levels (Figure 4b). L-NAME therapy alone did not have any effect on $NO_2^-+NO_3^-$ levels in the serum measured after the first or the second round (Figure 4a and b).

### *Effects of L-NAME and IL-2 therapy on the primary tumour size and number of lung metastatic nodules*

All forms of therapy (L-NAME alone at various doses, IL-2 alone or IL-2 + L-NAME) significantly ($P<0.05$) reduced the growth of primary tumours. The best responses were seen with L-NAME alone at 1 mg ml$^{-1}$, IL-2 alone or IL-2 + L-NAME (at various doses) (Figure 5). Addition of L-NAME to IL-2 therapy had only a transient (noted on day 14, at the end of the first round) beneficial effect on primary tumour growth (Figure 5), which was reproduced in another experiment (data not shown).

All therapies, except 1 mg ml$^{-1}$ L-NAME alone, significantly reduced the number of lung metastatic nodules. Reduction with IL-2 alone or IL-2 in combination with L-NAME was greater than with L-NAME alone (Figure 6).

### *Effects of D-NAME on IL-2-induced capillary leakage, NO production and anti-tumour effects*

$N^G$-nitro-D-arginine methyl ester (D-NAME) (1 mg ml$^{-1}$) had no significant effect on IL-2-induced pleural effusion, rise in water content in the spleen and kidney, or the reduction of the primary tumour size after the first round of therapies ($n=5$ per group, data not presented). Furthermore, D-NAME had no influence on IL-2-induced rise in the levels of $NO_2^-+NO_3^-$ in the serum and pleural effusion of the tumour-bearing mice after one round of therapies (data not presented). However, there was a minor reduction in IL-2-induced pulmonary oedema caused by addition of D-NAME. D-NAME alone (1 mg ml$^{-1}$) had no effect on the growth of the primary tumour (data not shown).

### Discussion

The results of the present study showed that oral administration of L-NAME, a potent inhibitor of NO synthesis, reduced the severity of IL-2-induced capillary leakage in some organs of tumour-bearing mice, without compromising the therapeutic benefits of IL-2. Furthermore, L-NAME therapy alone had significant anti-tumour effects.

We demonstrated that IL-2-induced pulmonary oedema, water retention in the spleen and rise in nitrite + nitrate levels (in the serum and pleural effusion) after the first round of therapy were all significantly reduced with addition of L-NAME. However, this addition did not affect the level of IL-2-induced pleural effusion or water retention in the kidney after one round of therapy. These results in tumour-transplanted mice are in general accord with those previously reported by us in healthy mice (Orucevic and Lala, 1995*a*). In both of these studies, two lines of evidence excluded the possibility that the therapeutic effects of L-NAME in reducing the water content of certain organs in IL-2-treated mice were due to L-NAME-induced reduction in water consumption (antidypsogenic effect): (1) we noted that higher doses of L-NAME (eg. 1 mg ml$^{-1}$), as well as the present dose of IL-2 led to a significant reduction in water consumption (up to 60% of pretreatment values at the end of the first round of therapy). However, the water consumption in IL-2-treated mice remained unaffected by the addition of L-NAME; (2) In spite of antidypsogenic effects, L-NAME therapy alone did not influence the water content of any organ in healthy mice. Furthermore, L-NAME is well recognised for its vasoconstrictive effects in healthy animals (Gross *et al.*, 1990). Since this effect alone did not influence

**Figure 4** $NO_3^-$ levels in the serum (**a**) after one round of therapy (**b**) after two rounds of therapy; and (**c**) pleural effusion after one round of therapy, measured as $NO_2^-$ after reduction. Data represent mean±s.e. ($n=3-5$, each done in duplicate). *Dose-dependent trend in reduction of $NO_3^-$ levels in the serum with addition of different doses of L-NAME to IL-2 after the first round of therapy. †Only the dose of 1 mg ml$^{-1}$ L-NAME caused significant ($P<0.05$) abrogation of IL-2-induced rise in $NO_3^-$ levels in the serum after the first round of therapies. ‡Addition of 1 mg ml$^{-1}$ L-NAME to IL-2 significantly ($P<0.05$) increased $NO_3^-$ levels in the serum after the second round of therapies. § IL-2-induced NO production in pleural effusion after the first round of therapy was significantly ($P<0.05$) reduced with addition of L-NAME.

**Figure 5** Growth of the primary tumour during IL-2 and L-NAME therapy, as given by the mean tumour volume on days 9, 14, 19 and 23. Data represent mean±s.e. (days 9 and 14: $n=10-20$; day 19 and day 23: $n=9-12$). After the first (day 14) or the second round (day 23) of therapies, IL-2 alone, or in combination with any dose of L-NAME, or L-NAME alone significantly ($P<0.05$) reduced the growth of the primary tumour. ○, Tumour control; ●, 0.1 mg ml$^{-1}$ L-NAME; ▽, 0.5 mg ml$^{-1}$ L-NAME; ▼, 1 mg ml$^{-1}$ L-NAME; □, IL-2; ■, IL-2+0.1 mg ml$^{-1}$ L-NAME; △, IL-2+0.5 mg ml$^{-1}$ L-NAME; ▲, IL-2+1 mg ml$^{-1}$ L-NAME.

the water content of any organ in healthy mice treated with L-NAME alone, the observed L-NAME-induced reduction of water content of the lungs and the spleen in IL-2-treated mice cannot be explained solely on the basis of generalised or selective vasoconstriction of normal vascular beds.

We suggest that the beneficial effects of L-NAME against IL-2-induced capillary leakage resulted from an abrogation of IL-2-induced NO overproduction (measured in the serum and pleural fluid) documented here, as well as in healthy tumour-free mice (Orucevic and Lala, 1995*a*). This abrogation occurred in spite of reported higher selectivity of L-NAME for binding and inactivation of the constitutive isoforms of NO synthase (NOS) enzymes in comparison with the inducible isoform (i-NOS) (Gross *et al.*, 1990; Dwyer *et al.*, 1991; Furfine *et al.*, 1993). For these reasons, further investigation is needed to identify the cellular source of NO as well as the NOS isoform(s) responsible for increased NO production after IL-2 therapy. This information may also indicate the relative contribution of haemodynamic (owing to eNOS) and other effects (owing to iNOS) of NO towards IL-2-induced capillary leakage.

At later stages of tumour growth (day 23), tumours themselves induced significant fluid retention in the lungs and the kidney, and the former was reduced with L-NAME therapy alone. It is likely that the increased fluid retention in the lungs was a direct result of pulmonary metastases due to either an increased leakiness of the capillaries supplying the metastatic foci, or an increased leakiness of the lung vasculature in the presence of metastasis. Thus a reduction in tumour-induced pulmonary oedema can be explained by the anti-metastatic effects of L-NAME (documented here) and/or a selective reduction in tumour blood flow (Andrade *et al.*, 1992) within the metastatic foci. IL-2 therapy alone, in spite of strong anti-metastatic effects, failed to reduce the

**Figure 6** Number of metastatic nodules in the lungs scored on day 23 after two rounds of IL-2 and L-NAME therapy ($n$=9–12). *Significant reduction ($P<0.05$) was noted with all therapies except with 1 mg ml$^{-1}$ L-NAME. □, mean±s.e.; ■, median.

water content of the lungs, probably because of additional IL-2-induced pulmonary oedema.

A second round of IL-2 therapy caused a milder degree of capillary leakage (fluid retention in the spleen, but not the lungs, kidney or the pleural cavities), which was not ameliorated by addition of L-NAME. In parallel, there was no significant change in NO production in most groups receiving IL-2 or the combination therapy. While this association may reinforce the role of NO in IL-2-induced capillary leakage, the reasons for a resistance of healthy mice (Orucevic and Lala, 1995*b*) as well as tumour-bearing mice (this study) to the induction of pleural effusion by the second round of IL-2 remains unclear. Identification and cellular localisation of NOS isoforms in the treated mice may resolve this issue. The expression of iNOS is known to vary from tissue to tissue and within a given tissue under different circumstances (Nussler and Billiar, 1993). Thus, it is possible that our second round of IL-2 did not induce iNOS in the pleural vessels, which consequently remained resistant to leakage. The so called 'tolerance' to IL-2 toxicity after the initial round reported by our laboratory in the human (Mertens *et al.*, 1993) and a milder fall in blood pressure after the second course of IL-2 reported by another laboratory (Hibbs *et al.*, 1992) might be similarly explained.

We found that anti-tumour effects of IL-2 were not compromised with addition of L-NAME and that L-NAME therapy alone had anti-tumour effects. These findings were similar to our previous observations with another NOS inhibitor, NMMA (Orucevic and Lala, 1993). The observed anti-tumour effects of NOS inhibitors in our tumour model are in accord with those documented in a rat model (Kennovin *et al.*, 1993, 1994).

Mechanisms responsible for the anti-tumour effects of NOS inhibitors in the present as well as other studies remain to be fully investigated. They may include a reduction in the tumour blood flow, tumour angiogenesis, tumour invasiveness and an abrogation of NO-induced immunosuppression in the host. Evidence exists for some of the above. Kennovin *et al.* (1993, 1994) observed that the anti-tumour effects were not sustained after withdrawal of chronic L-NAME therapy of tumour-bearing rats. For this and other reasons, they postulated that the therapeutic effects were probably due to a reduction in tumour blood flow, caused by a selective constriction of the tumour vasculature owing to an inhibition of iNOS activity. Indeed, NO production by experiment tumours in mice has been implicated in maintaining tumour blood flow in the neovasculature (Buttery *et al.*, 1993), and its blockade with L-NAME has been shown to selectively reduce flow in tumour-associated neovasculature (Andrade *et al.*, 1992) and to cause a severe tumour hypoxia (Wood *et al.*, 1993).

There is a growing number of reports on the opposing roles of NO on the immune system that may influence tumour growth. While NO may be required for tumoricidal function of certain effector cells, excessive NO production can also suppress lymphocyte activation. It has been shown that activated murine macrophages synthesise NO from L-arginine (Stuehr and Marletta, 1985), which may partly mediate the cytotoxic activity of these cells against tumour cells and bacteria (Lancaster and Hibbs, 1990). Mills *et al.* (1992) reported that tumour growth in the peritoneal cavity of mice was associated with a marked decline in the production of NO by intra-tumour macrophages. On the other hand, murine macrophages have been shown to down-regulate lymphocyte activation by an NO-dependent mechanism (Hoffman *et al.*, 1990; Albina *et al.*, 1991), which may compromise the tumoricidal function of lymphocytes. Indeed, NO has been implicated in the tumour-induced immunosuppression in rats (Lejeune *et al.*, 1994) and high NO synthase activity has been correlated with the degree of malignancy (Thomsen *et al.*, 1994).

In a preliminary study (A Orucevic and PK Lala, unpublished), we found an improvement in IL-2-induced LAK cell function *in vivo* with addition of L-NAME in tumour-bearing mice after the first round of therapy. This may explain our findings of a transient improvement of IL-2-induced retardation of tumour growth by addition of L-NAME after the first round of therapy (present study). Although L-NAME alone did not significantly change serum levels of nitrite+nitrate, we demonstrated that L-NAME-mediated reduction of tumour size was related to NO pathway, since treatment of mice with D-NAME, an amino acid incapable of inhibition of NO generation (Ialenti *et al.*, 1993), failed to influence tumour growth rate (data not shown). We are currently investigating the identity of the NOS isoforms and the cellular source of NO in our tumour model.

In summary, L-NAME therapy had an anti-tumour effect when given alone. In combination with IL-2, L-NAME reduced the severity of IL-2-induced capillary leakage in some organs after the first round of therapy and did not compromise anti-tumour effects of IL-2 therapy. Thus, L-NAME could be a valuable adjunct to IL-2-based immunotherapy of cancer.

### Abbreviations

IL-2, interleukin 2; LAK, lymphokine-activated killer; NK, natural killer; IFN, interferon; TNF, tumour necrosis factor; NO, nitric oxide; NMMA, $N^G$-methyl-L-arginine; L-NAME, $N^G$-nitro-L-arginine methyl ester; $NO_2^-$, nitrite; $NO_3^-$, nitrate; U, Cetus units; NOS, nitric oxide synthase.

### Acknowledgements

This study was supported by grants from the National Cancer Institute of Canada with funds from the Canadian Cancer Society to PKL, and a Fellowship from the Medical Research Council of Canada to AO. We appreciate the helpful discussion of this paper with Dr Salvador Moncada (The Wellcome Research Laboratories, Beckenham, Kent, UK).

### References

ALBINA JE, ABATE JA AND HENRY WL JR. (1991). Nitric oxide production is required for murine resident peritoneal macrophages to suppress mitogen-stimulated T cell proliferation: role of IFN$\gamma$ in the induction of nitric oxide-synthesising pathway. *J. Immunol.*, **147**, 144–148.

AMADOR J-F, VAZQUEZ AM, CABRERA L, BARRAL AM, GENDELMAN R AND JONDAL M. (1991). Toxic effects of interleukin-2-activated lymphocytes on vascular endothelial cells. *Nat. Immun. Cell Growth Regul.*, **10**, 207–215.

ANDRADE SP, HART IR AND PIPER PJ. (1992). Inhibitors of nitric oxide synthase selectively reduce flow in tumour-associated neovasculature. *Br. J. Pharmacol.*, **107**, 1092–1095.

ARONSON FR, LIBBY P, BRANDON EP, JANICKA MW AND MIER JW. (1988). IL-2 rapidly induces natural killer cell adhesion to human endothelial cells. *J. Immunol.*, **141**, 158–163.

BAGULEY BC, CALVELEY SB, CROWE KK, FRAY LM, O'ROURKE SA AND SMITH GP. (1989). Comparison of the effects of flavone acetic acid, fostriecin, homoharringtone and tumour necrosis factor $\alpha$ on colon 38 tumours in mice. *Eur. J. Cancer Clin. Oncol.*, **25**, 263–269.

BRODT P, PARHAR RS, SANKER P AND LALA PK. (1985). Studies on clonal heterogeneity in two spontaneously metastasising mammary carcinomas of recent origin. *Int. J. Cancer*, **35**, 265–273.

BUTTERY LDK, SPRINGALL DR, ANDRADE SP, RIVEROS-MORENO V, HART I, PIPER PJ AND POLAK JM. (1993). Induction of nitric oxide synthase in the neo-vasculature of experimental tumours in mice. *J. Pathol.*, **171**, 311–319.

DAVISON W AND WOOF C. (1978). Comparison of different forms of cadmium as reducing agents for the batch determination of nitrate. *Analyst*, **103**, 403–406.

DUTCHER JP, CREEKMORE S, WEISS GR, MARGOLIN K, MARKOWITZ AB, ROPER M, PARKINSON D, CIOBANU N, FISHER RI, BOLDT DH, DOROSHOW JH, RAYNER AA, HAWKINS M AND ATKINS M. (1989). A phase II study of interleukin-2 and lymphokine activated killer (LAK) cells in patients with metastatic malignant melanoma. *J. Clin. Oncol.*, **7**, 477–485.

DWYER MA, BREDT DS AND SNYDER SH. (1991). Nitric oxide synthase: irreversible inhibition by L-$N^G$-nitroarginine in brain *in vitro* and *in vivo*. *Biochem. Biophys. Res. Commun.*, **176**, 1136–1141.

ETTINGHAUSEN SE, PURI RK AND ROSENBERG SA. (1988). Increased vascular permeability in organs mediated by the systemic administration of lymphokine-activated killer cells and recombinant interleukin-2 in mice. *J. Natl. Cancer Inst.*, **80**, 177–188.

FAGGIONI R, ALLAVENA P, CANTONI L, CARELLI M, DEMITRI MT, DELGADO R, GATTI S, GNOCCHI P, ISETTA AM, PAGANIN C, ECHTENACHER B, ALBINI E AND GHEZZI P. (1994). Mechanisms of interleukin-2-induced hydrothoraxy in mice: protective effect of endotoxin tolerance and dexamethasone and possible role of reactive oxygen intermediates. *J. Immunother.*, **15**, 194–201.

FISHER RI, COLTMAN CA, DOROSHOW JA, RAYNER AA, HAWKINS MJ, MIER JW, WIERNIK P, MCMANNIS JD, WEISS GR, MARGOLIN KA, GEMLO BT, HOTH DF, PARKINSON DR AND PAIETTA E. (1988). A phase II study of interleukin-2 and lymphokine activated killer cells (LAK) in metastatic renal cancer. *Ann. Intern. Med.*, **108**, 518–523.

FURFINE ES, HARMON MF, PATH JE AND GARVEY EP. (1993). Selective inhibition of constitutive nitric oxide synthase by L-$N^G$-nitroarginine. *Biochemistry*, **32**, 8512–8517.

GREEN LC, WAGNER DA, GLOGOWSKI J, SKIPPER PL, WISHNOK JS AND TANNENBAUM SR. (1982). Analysis of nitrate, nitrite, and [$^{15}$N]nitrate in biological fluids. *Anal. Biochem.*, **126**, 131–138.

GROSS SS, STUEHR DJ, AISAKA K, JAFFE EA, LEVI R AND GRIFFITH OW. (1990). Macrophage and endothelial cell nitric oxide synthesis: cell-type selective inhibition by $N^G$-nitroarginine and $N^G$-methylarginine. *Biochem. Biophys. Res. Commun.*, **170**, 96–102.

HIBBS JB JR, WESTENFELDER C, TAINTOR R, VAVRIN Z, KABLITZ C, BARANOWSKI RL, WARD JH, MENLOVE RL, MCMURRY MP, KUSHNER JP AND SAMLOWSKI WE. (1992). Evidence for cytokine-inducible nitric oxide synthesis from l-arginine in patients receiving interluekin-2 therapy. *J. Clin. Invest.*, **89**, 867–877.

HOFFMAN RA, LANGREHR JM, BILLIAR TR, CURRAN RD AND SIMMONS RL. (1990). Alloantigen-induced activation of rat splenocytes is regulated by the oxidative metabolism of L-arginine. *J. Immunol.*, **145**, 2220–2226.

IALENTI A, MONCADA S AND DI ROSA M. (1993). Modulation of adjuvant arthritis by endogenous nitric oxide. *Br. J. Pharmacol.*, **110**, 701–706.

KAHALEH MB, SMITH EA, SOMA Y AND LEROY EC. (1988). Effect of lymphotoxin and tumour necrosis factor *in vivo* and their prevention by cyclooxygenase inhibitors. *Clin. Immunol. Immunopath.*, **49**, 261–272.

KELM M, FEELISCH M, GRUBE R, MOTZ W AND STAUER BE. (1992). Metabolism of endothelium-derived nitric oxide in human blood. In *The Biology of Nitric Oxide*, Moncada S, Marletta MA, Hibbs JB, Jr and Higgs EA. (eds) pp.319–322. Portland Press: London.

KENNOVIN GD, HIRST DJ AND FLITNEY FW. (1993). Oral $N^G$-nitro-L-arginine methyl ester (L-NAME) administration slows the growth of malignant tumours in rodents (abstract). *Endothelium*, **1**, s52.

KENNOVIN GD, HIRST DG, STRATFORD MRL AND FLITNEY FW. (1994). Inducible nitric oxide synthase is expressed in tumour-associated vasculature: inhibition retards tumour growth *in vivo*. In *Biology of Nitric Oxide Vol 4. Enzymology, Biochemistry and Immunology*, Moncada S, Feelisch M, Busse R and Higgs EA (eds) pp.473–479. Portland Press: London.

LALA PK AND PARHAR RS. (1993). Eradication of sponaneous and experimental adenocarcinoma metastases with chronic indomethacin and intermittent IL-2 therapy. *Int. J. Cancer*, **54**, 677–684.

LALA PK, PARHAR RS AND SINGH P. (1986). Indomethacin therapy abrogates the prostaglandin-mediated suppression of natural killer activity in tumour-bearing mice and prevents tumour metastasis. *Cell. Immunol.*, **99**, 108–118.

LANCASTER JR JR AND HIBBS JB JR. (1990). EPR demonstration of iron-nitrosyl complex formation by cytotoxic activated macrophages. *Proc. Natl Acad. Sci. USA*, **87**, 1223–1227.

LEJEUNE P, LAGADEC P, ONIER N, PINARD D, OHSHIMA H AND JEANNIN J-F. (1994). Nitric oxide involvement in tumour-induced immunosuppression. *J. Immunol.*, **152**, 5077–5083.

MARGOLIN KA, RAYNER AA, HAWKINS MJ, ATKINS MB, DUTCHER JP, FISHER RI, WEISS GR, DOROSHOW JH, JAFFE HS, ROPER M, PARKINSON DR, WIERNIK PH, CREEKMORE SP AND BOLDT DH. (1989). Interleukin-2 and lymphokine-activated killer cell therapy of solid tumours: analysis of toxicity and management guidelines. *J. Clin. Oncol.*, **7**, 486–498.

MERTENS WC, BRAMWELL VHC, BANERJEE D, GWADRY-SRIDHAR F, AL-MUTTER N, PARHAR RS AND LALA PK. (1993). Chronic oral indomethacin and ranitidine with intermittent continuous infusion interleukin-2 in advanced renal cell carcinoma. *Cancer Biotherapy*, **8**, 229–233.

MILLS CD, SHEARER J, EVANS R AND CALDWELL MD. (1992). Macrophage arginine metabolism and inhibition or stimulation of cancer. *J. Immunol.*, **149**, 2709–2714.

MONCADA S AND HIGGS A. (1993). The L-arginine nitric oxide pathway. *N. Engl. J. Med.*, **329**, 2002–2012.

MONTESANO R, ORCI L AND VASSALLI P. (1985). Human endothelial cell cultures: Phenotypic modulation by leukocyte interleukins. *J. Cell. Physiol.*, **122**, 424–434.

NUSSLER AK AND BILLIAR TR. (1993). Inflammation, immunoregulation, and inducible nitric oxide synthase. *J. Leuk. Biol.*, **54**, 171–178.

OCHOA JB, CURTI B, PEITZMAN AB, SIMMONS RL, BILLIAR TR, HOFFMAN R, RAULT R, LONGO DL, URBA WJ AND OCHOA AC. (1992). Increased circulating nitrogen oxides after human tumour immunotherapy: correlation with toxic hemodynamic changes. *J. Natl Cancer Inst.*, **84**, 864–867.

ORUCEVIC A AND LALA PK. (1992). Effects of $N^G$-methyl-L-arginine and indomethacin on IL-2 induced pulmonary oedema and pleural effusion (abstract). *Proc Am. Assoc. Cancer Res.*, **33**, 332.

ORUCEVIC A AND LALA PK. (1993). Effects of $N^G$-methyl-L-arginine and indomethacin on IL-2 induced capillary leakage in tumour-bearing mice (abstract). *Proc. Am. Assoc. Cancer Res.*, **34**, 459.

ORUCEVIC A AND LALA PK. (1994). Effects of $N^G$-nitro-L-arginine, a nitric oxide inhibitor, on IL-2 induced capillary leak syndrome (abstract). *Proc. Canad. Fed. Biol. Soc.*, 37th annual meeting, 117.

ORUCEVIC A AND LALA PK. (1995*a*). $N^G$-nitro-L-arginine methyl ester, an inhibitor of nitric oxide synthesis, ameliorates interlukin-2-induced capillary leak syndrome in healthy mice. *J. Immunother.*, in press.

ORUCEVIC A AND LALA PK. (1995*b*). Effects of $N^G$-methyl-L-Arginine, an inhibitor of nitric oxide synthesis, on IL-2 induced capillary leakage and anti-tumor responses in healthy and tumor bearing mice. (submitted).

PARKINSON DR, FISHER RI, RAYNER AA, PAIETTA E, MARGOLIN KA, WEISS GR, MIER JW, SZNOL M, GAYNOR ER, BAR MH, GUCALP R, BOLDT DH, MILLS B AND HAWKINS MJ. (1990). Therapy of renal cell carcinoma with interleukin-2 and lymphokine-activated killer cells: Phase II experience with a hybrid bolus and continuous infusion interleukin-2 regimen. *J. Clin. Oncol.*, **8,** 1630-1636.

PURI RK, TRAVIS WD AND ROSENBERG SA. (1989). Decrease in interleukin 2-induced vascular leakage in the lungs of mice by administration of recombinant interleukin 1$\alpha$ *in vivo*. *Cancer Res.*, **49,** 969-976.

ROSENBERG SA. (1989). Clinical immunotherapy studies in the surgery branch of the US National Cancer Institute. *Cancer Treat. Rev.*, **16** (Suppl A), 115-121.

ROSENBERG SA, MULE JJ, SPIESS PJ, REICHART CM AND SCHWARZ SL. (1985). Regression of established pulmonary metastasis and subcutaneous tumour mediated by systemic administration of high dose recombinant interleukin 2. *J. Exp. Med.*, **161,** 1169-1188.

ROSENBERG SA, LOTZE MT, MUUL LM, CHANG AE, AVIS FP, LEITMAN S, LINEHAM M, ROBERTSON CN, LEE RE, RUBIN JT, SEIPP CA, SIMPSON C AND WHITE DE. (1987). A progress report on the treatment of 157 patients with advanced cancer using lymphokine-activated killer cells and interleukin-2 or high-dose interleukin-2 alone. *N. Engl. J. Med.*, **316,** 889-897.

ROSENSTEIN M, ETTINGHAUSEN SE AND ROSENBERG SA. (1986). Extravasation of intravascular fluid mediated by the systemic administration of recombinant interleukin 2. *J. Immunol.*, **137,** 1735-1742.

SAARLOOS MN, KHOO NKS AND LALA PK. (1992). Effects of cancer immunotherapy with indomethacin and interleukin-2 on murine hemopoietic stem cells. *Cancer Res.*, **52,** 6452-6462.

SIEGEL JP AND PURI PK. (1991). Interleukin-2 toxicity. *J. Clin. Oncol.*, **9,** 694-704.

STUEHR DJ AND MARLETTA MA. (1985). Mammalian nitrate biosynthesis: mouse macrophages produce nitrite and nitrate in response to *Escherichia coli* lipopolysaccharide. *Proc. Natl Acad. Sci. USA*, **82,** 7738-7742.

THOMSEN LL, LAWTON FG, KNOWLES RG, BEESLEY JE, RIVEROS-MORENO V AND MONCADA S. (1994). Nitric oxide synthase activity in human gynecological cancer. *Cancer Res.*, **54,** 1352-1354.

WELBOURN R, GOLDMAN G, KOBZIK L, VALERI CR, HECHTMAN HB AND SHEPRO D. (1991). Attenuation of IL-2-induced multisystem organ oedema by phalloidine and antamanide. *J. Appl. Physiol.*, **70,** 1364-1368.

WOOD PJ, STRATFORD IJ, ADAMS GE, SZABO C, THIEMERMANN C AND VANE JR. (1993). Modification of energy metabolism and radiation response of murine tumour by changes in nitric oxide availability. *Biochem. Biophys. Res. Commun.*, **192,** 505-510.

ZAR JH. (1974). *Biostatistical Analysis*. Prentice-Hall: Englewood Cliffs, NJ.